# STAT6-IP–dependent inhibition of type 2 innate and Th2 adaptive immunity in the murine lung

**DOI:** 10.1093/immhor/vlag012

**Published:** 2026-03-29

**Authors:** Haya Aldossary, Rami Karkout, Véronique Gaudreault, Lydia Labrie, Jichuan Shan, Elizabeth D Fixman

**Affiliations:** Meakins-Christie Laboratories, Research Institute of McGill University Health Centre, Montréal, QC, Canada; Meakins-Christie Laboratories, Research Institute of McGill University Health Centre, Montréal, QC, Canada; Meakins-Christie Laboratories, Research Institute of McGill University Health Centre, Montréal, QC, Canada; Meakins-Christie Laboratories, Research Institute of McGill University Health Centre, Montréal, QC, Canada; Meakins-Christie Laboratories, Research Institute of McGill University Health Centre, Montréal, QC, Canada; Meakins-Christie Laboratories, Research Institute of McGill University Health Centre, Montréal, QC, Canada

**Keywords:** alarmins, dendritic cells, group 2 innate lymphoid cells, type 2 innate immunity, Th2 adaptive immunity

## Abstract

Type 2 airway inflammation is one of the main characteristics of allergen-induced asthma. Evidence from animal studies supports a model in which inhalation of allergens triggers epithelial cell release of alarmin cytokines, including IL-33, which activate a number of innate cells in the lung, including group 2 innate lymphoid cells (ILC2s), which produce large amounts of IL-13 and IL-5, to amplify allergic inflammation. Amongst other activities, ILC2-derived IL-13 promotes DC migration to the lung draining mediastinal lymph nodes (MLNs). Our published data indicate that topical administration of an immunomodulatory peptide, STAT6-IP, at the time of antigen priming inhibits T helper 2 adaptive immunity in murine models of asthma, at least in part, through inhibition of dendritic cells (DCs). In this study, we sought to clarify inhibitory activity of STAT6-IP toward DC responses in the lung and the lung draining MLNs induced by IL-33 and ovalbumin (OVA). Our data show that STAT6-IP reduced expansion of total and IL-13–producing ILC2s in OVA/IL-33–treated mice. STAT6-IP also inhibited OVA/IL-33–induced recruitment to and activation of lung DCs, which in turn reduced DC migration and CD4^+^ Th2 differentiation in the lung MLNs. When challenged several weeks later with OVA, allergic inflammatory responses, including airway hyperresponsiveness, were reduced in STAT6-IP–treated mice. STAT6-IP retained inhibitory activity whether delivered before or after OVA/IL-33 and activity coincided with expansion of IL-13–producing ILC2s. Altogether, our findings provide insight into mechanisms by which STAT6-IP interacts with innate immune cells of the lung to reduce maladaptive type 2 innate and T helper 2 adaptive immunity.

## Introduction

The most common features associated with allergic asthma in humans, and replicated in murine models, primarily in Balb/c and C57Bl/6 mice, include airway hyperreactivity (AHR), accompanied by increases in eosinophils, IgE, and mucus production.^1^ Following exposure to environmental triggers, including allergens, lung epithelial cells release alarmin cytokines, including IL-33, thymic stromal lymphopoietin, and IL-25, which induce responses in a number of immune cells.^2^ IL-33, a member of the IL-1 cytokine family, promotes both innate and adaptive immunity,^3^ inducing responses in a wide range of cells, including dendritic cells (DCs), eosinophils, basophils, mast cells, natural killer T cells, CD4^+^ and CD8^+^ T cells, macrophages, and group 2 innate lymphoid cells (ILC2s).

Abundant data from murine models demonstrate that activation of ILC2s promotes type 2 innate inflammation in the lung.^1^ The contribution of ILC2s in initiating and amplifying T helper 2 (Th2) adaptive immunity in murine models has also been highlighted.^4–6^ Upon activation, ILC2s produce large amounts of the type 2 cytokines, most prominently IL-13 and IL-5, which in turn amplify allergic inflammation. In particular, there is evidence that IL-13 produced by ILC2s, in response to house dust mite, ovalbumin (OVA) plus IL-33, or the protease allergen papain, induces migration of DCs to the lung draining mediastinal lymph nodes (MLNs), where they induce Th2 differentiation.^7^^,^^8^ In the absence of IL-13–producing ILC2s, numbers of activated DCs expressing CD40 as well as Th2 differentiation in the MLNs are dramatically reduced.^8^ Although the precise mechanisms that regulate IL-13–dependent DC migration to the MLNs are unclear, several factors influence lung DC migration, first and foremost upregulation of CCR7, which promotes DC migration toward the T cell zone in the MLNs.^9^

Our data demonstrate that intranasal administration of the STAT6-IP immunomodulatory peptide, but not a negative control peptide, STAT6-CP (which differs by 1 amino acid), at the time of antigen priming inhibits maladaptive Th2 inflammation, including AHR and recruitment of eosinophils to the airways, in both asthma^10^ and respiratory syncytial virus infection models.^11^^,^^12^ Moreover, STAT6-IP reduces the frequency of OX40L expressing CD11b^+^CD11c^+^ lung DCs in mice treated with OVA and muramyl dipeptide (MDP), a Nod2 agonist that promotes Th2 differentiation.^10^ STAT6-IP delivery at the time of OVA/MDP priming reduces allergic airway inflammatory responses induced upon secondary OVA challenge several weeks later. More recently, we have shown that STAT6-IP, but not control STAT6-CP, inhibits IL-33–induced ILC2 expansion and cytokine production, acting as a potent inhibitor of type 2 innate inflammation.^13^

In this study, we sought to clarify how STAT6-IP affects DC responses in the lung and the MLNs induced by IL-33 in the presence of OVA. Our data show that STAT6-IP inhibited OVA/IL-33–induced recruitment and activation of both conventional DCs (cDCs) and monocyte-derived DCs (moDCs), which in turn reduced DC migration to the MLNs and subsequent CD4^+^ Th2 differentiation. Because ILC2-derived IL-13 can promote DC migration to the MLNs,^8^ we also quantified ILC2 responses. Both total as well as IL-13–expressing ILC2s were reduced in mice treated with STAT6-IP. Though most potent when delivered before OVA/IL-33, STAT6-IP retained inhibitory activity even when delivered afterward, coinciding with inhibition of ILC2 expansion and cytokine production. Consistent with these data, STAT6-IP administration at the time of antigen priming reduced allergic inflammatory responses, including AHR, mucus production, and eosinophil influx into the lung following OVA challenge several weeks later. Our findings provide evidence that STAT6-IP targets innate immune cells, including ILC2s and DCs, to disrupt the induction of Th2 adaptive immunity.

## Methods

### Mice

Wild-type Balb/c mice were bred in house in the Animal Resource Division at the Research Institute of McGill University Health Centre and male mice were used at ages 5 to 8 wk. All animal studies were approved by the McGill University Animal Care Committee and performed following guidelines of the Canadian Council on Animal Care.

### In vivo stimulation and STAT6-IP delivery

Mice were briefly anesthetized with isoflurane, followed by intranasal delivery of IL-33 (0.5 µg) and OVA (50 µg) in a volume of 30 µL on day 1 and day 2. As a negative control, OVA was delivered alone, as previously.^10^^,^^14^^,^^15^ IL-33 was purchased from Thermo Fisher Scientific and OVA was purchased from Worthington Biochemical Corp. For experiments to examine DCs or ILC2s, saline or STAT6-IP (100 µg) was given intranasally 1 d before and 1 h before each OVA/IL-33 administration (IP-OVA/IL-33). Another group was treated with STAT6-IP 24 h and 48 h after the second OVA/IL-33 delivery (OVA/IL-33-IP). Mice were euthanized on day 5, and lungs and MLNs were harvested for ex vivo analysis (flow cytometry and quantitative real-time polymerase chain reaction [qRT-PCR]). To assess CD4^+^ T cell differentiation in the MLNs, mice were treated as previous and euthanized on day 8 in order to collect MLNs for flow cytometry analysis.

For OVA challenge experiments, mice were treated with IL-33 (0.05 µg), and/or OVA (50 µg) as previous. For the IP-OVA/IL-33 group, STAT6-IP was delivered as previous. The IP-A group received STAT6-IP for 4 consecutive days after the second OVA/IL-33 delivery. Mice were then allowed to rest for a period of 4 to 5 wk, after which they were challenged with OVA (10 μg) daily for each of 4 d before sacrifice 48 h after the last challenge in order to harvest lungs and MLNs. Blood was collected following cardiac puncture.

### Tissue collection and flow cytometry

Lung tissue was collected in RPMI medium, minced, and incubated in a 1 mL enzymatic solution of RPMI with DNase I (200 μg/mL; Sigma-Aldrich), Liberase (100 μg/mL; Roche), hyaluronidase 1a (1 mg/mL; Life Technologies), and collagenase XI (250 μg/mL; Life Technologies), in a 12-well plate. The plates were incubated for 35 min at 37 °C, followed by adding 1 mL of RPMI + 5% fetal bovine serum (FBS) + 1% penicillin/streptomycin to stop the enzymatic digestion.^16^^,^^17^ Red blood cells were lysed by adding 3 mL of ACK lysis buffer, followed by 2 washes with RPMI + 5% FBS + 1% penicillin/streptomycin. Then, the cells were filtered, counted, and diluted using RPMI + 5% FBS to obtain 2 × 10^6^, 2 × 10^6^, 1.25 × 10^6^, and 1 × 10^6^ cells for DC, ILC2, T cell, and eosinophil staining, respectively.

MLNs were collected and processed, and the single-cell suspension was prepared for staining. All cells for flow cytometry analysis were first washed with phosphate-buffered saline (PBS) before being stained with eFluor780 Viability Dye (eBioscience) and incubated at 4 °C in the dark. After 20 min, cells were washed and incubated for 5 min with anti‐mouse CD16/CD32 (Fc block) (BD Biosciences).

To characterize DC populations, and to examine the expression of CD40 costimulatory molecule and CCR7 on lung and MLN DCs, cells were stained with Alexa Fluor 700-CD45, FITC-F4/80, APC-CD11c, BV510-MHCII, BV605-CD11b, PeCy7-CD103, PerCP-eFluor710-FceR1, BV650-B220, BUV395-CD40, BV786-CCR7, and BUV737-CD8a along with an exclusion channel containing a combination of PE-conjugated antibodies to CD19, CD3e, CD49b, and Ly6G.

Lung eosinophils were stained with the following antibodies: BUV395-CD45.2, Alexa Fluor 700-Ly6G, AF488-CD11c, PeCy7-CD11b, PE-Siglec F, and APC-F4/80.

For ILC2 and T cell intracellular cytokine staining, lung cells were plated in duplicate in a low-adherent round-bottom 96-well plate. Then, plates were centrifuged at 400 *g* for 10 min. For T cell staining, a cocktail of 0.5 µg/mL PMA and 1 µg/mL ionomycin in the presence of 0.133 µL of GolgiStop (BD Cytofix/Cytoperm kit; BD Biosciences) in 200 µL of RPMI + 10% FBS was added to each well. To stain ILC2s, 0.133 µL of GolgiStop (BD Cytofix/Cytoperm kit) in 200 µL of RPMI + 10% FBS was added to each well. Then, plates were incubated at 37 °C for 4 h. After incubation, plates were centrifuged at 400 *g* for 10 min and cells were resuspend in 100 µL of PBS and duplicate wells were combined and extracellular staining was conducted. ILC2s were stained with BUV395-CD45.2, eF450-Thy1.2, PeCy7-CD127, PerCP-eF710-ST2, BV605-KLRG1, BV510-MHCII, and a combination of PE-conjugated antibodies that recognize CD3e, CD11c, CD11b, CD49b, CD45R, TCRyD, Ly6G, and FceR1a, while T cells were stained with BUV395-CD45.2, V500-CD3, FITC-CD4, and PerCP-Cy5.5-CD8. Afterward, cells were washed and fixed overnight in 100 µL of IC fixation buffer (eBioscience).

The next day, cells were washed with 100 µL of BD PermWash buffer per well. Then, 200 µL of BD PermWash was added for each well and cells were incubated at 4 °C for 15 min, after which plates were centrifuged at 400 *g* for 10 min. ILC2s were stained with a cocktail of AF488-IL-13 and APC-IL-5, and T cells were stained with PE-IL-13, BV421-IL-5, and APC-IL-4 prepared in 100 µL of cold BD Perm/Wash buffer per sample. Cells were incubated in the dark for 45 min at 4 °C, after which cells were washed twice with Perm buffer and one time with 200 µL of PBS. Finally, cells were resuspended in 100 µL PBS for acquisition.

In all flow panels, fluorescence minus one controls were used to define and gate positive populations. More information about the antibodies can be found in Tables S1 to S3. Cells were acquired with BD LSRFortessa or BD LSRFortessa X-20 (Immununophenotyping Core Facility, RI-MUHC) flow cytometer. Analysis was completed with FlowJo V10 (FlowJo LLC).

### RNA extraction and qRT-PCR

qRT-PCR was performed as described previously.^18^ The lower lobe of the right lung was collected for qPCR, and immediately frozen in liquid nitrogen, then stored at −80 °C. RNA was extracted using phenol-chloroform with TRIzol reagent (Invitrogen), after which RNA quantity and purity were assessed by UV spectroscopy using the NanoDrop 2000 (Thermo Fisher Scientific). The integrity of extracted RNA was assessed using the bleach gel method.^19^ A total of 1 μg of RNA was used for cDNA generation using Maxima H Minus First Strand cDNA Synthesis Kit (Thermo Fisher Scientific) with a DNase step, according to the manufacturer’s instructions. The quality of cDNA and primer efficiency were validated with template standard curves. Gene-specific primer sequences were as follows: *CCL2*, F: 5′-GAAGGAATGGGTCCAGACAT-3′, R: 5′-ACGGGTCAACTTCACATTCA-3′; *CCL20*, F: 5′-GTACTGCTGGCTCACCTCTG-3′, R: 5′-CTTCATCGGCCATCTGTCTTGTG-3′; *b-actin*, F: 5′-AGCCATGTACGTAGCCATCC-3′; R: 5′-CTCTCAGCTGTGGTGGTGAA-3′; *IL-13*, F: 5′-TGGGTCCTGTAGATGGCATTG-3′, R: 5′-AGACCAGACTCCCCTGTGCA-3′; *Muc5ac*, F: 5′-CAGCCGAGAGGAGGGTTTGATCT-3′, R: 5′-AGTCTCTCTCCGCTCCTCTCAAT-3′.

Genes were amplified using the StepOnePlus real-time PCR system and the Power Up SYBR green master mix from Thermo Fisher Scientific. Gene fold change expression was normalized to β-actin and differences were expressed as relative fold change to the control group using the ΔΔCt method.

### OVA‐specific IgE

High binding costar 96 well plates were coated with 100 µL of 2 µg/mL anti-IgE (eBioscience; clone R35-72), diluted in 100 mM sodium carbonate/bicarbonate buffer and plates were incubated overnight at 4 °C. Plates were washed 5 times with PBS‐Tween 20 (0.05%) and blocked using assay diluent (200 µL/well). After 1 hour of incubation, diluted standard and serum samples were added and incubated overnight at 4 °C. The next day, plates were washed with PBS‐Tween 20 (0.05%) 5 times, and 100 µL of 2 µg/mL of OVA conjugated to horseradish peroxidase (BD Biosciences) was added and incubated for 1 h. Afterward, the plates were washed 7 times, followed by adding 100 µL of tetramethylbenzadine to each well. Plates then covered and incubated in the dark, at room temperature, under mild agitation, for 30 min. Finally, and prior to detection, the reaction was stopped with 50 µL of 1M H_3_PO_4_.

### Lung function analysis and histology

Pulmonary function was assessed as described previously.^10^^,^^18^ Briefly, mice were anesthetized with xylazine and sodium pentobarbital and paralyzed with pancuronium bromide. Then, total lung elastance and resistance in response to increasing doses of inhaled methacholine were measured with the flexiVent small-animal ventilator (Scireq). Baseline respiratory system resistance and maximal resistance to increasing doses of nebulized methacholine were measured. For histology, lungs were perfused with 1× PBS followed by injecting 10% formalin through the trachea until the lungs were fully inflated. Then, entire lungs were collected and placed in formalin. Afterward, lungs were embedded in paraffin, and 4 μm sections were cut and mounted on glass slides. Lung sections were stained with hematoxylin and eosin or periodic acid–Schiff. Zeiss Axio Imager M2 microscope was used to take pictures and Zeiss Zen software was used to build the figure. Images were quantified by an individual blinded to the groups, scoring inflammation and mucus production from 3 to 6 randomly chosen airways per mouse. A value of “0” indicated no inflammation or no mucus. The most abundant tissue inflammation and mucus assigned scores of 2 and 5, respectively.

### Statistical analysis

Data analyses and graphs were generated using Prism version 9 (GraphPad Software). Data were analyzed using 1- or 2-way analysis of variance followed by multiple comparisons using Tukey’s post hoc test. A *P* value <0.05 was considered significant. Outcomes are presented as mean ± SEM. Grubb’s test with α = 0.05 was used to remove outliers.

## Results

### STAT6-IP inhibits OVA/IL-33–induced lung DC recruitment and activation, reducing their migration to the lung MLNs

We previously showed that delivery of STAT6-IP at the time of antigen priming reduces DC responses and Th2 adaptive immunity in an OVA model of allergic airways disease.^10^ Here, we sought to better understand how STAT6-IP interacts with innate immune cells in the lung and to define which DC subsets were targeted by STAT6-IP to reduce acute Th2 differentiation and Th2 adaptive immunity. We assessed effects of STAT6-IP on activation and migration of different migratory and inflammatory DC subsets. We also examined whether STAT6-IP retained inhibitory activity when delivered after administration of OVA/IL-33.

**Figure 1:**
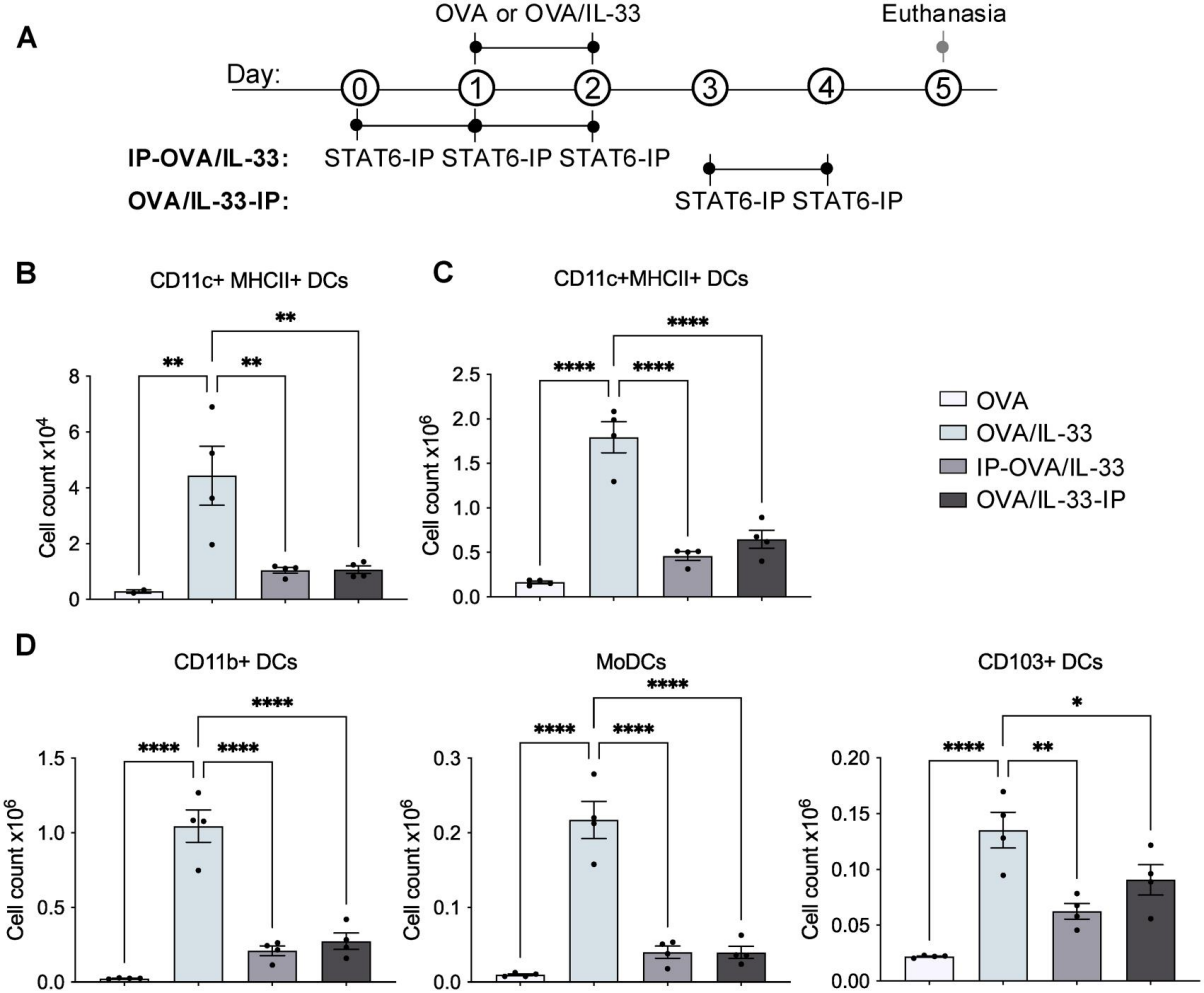
STAT6-IP reduces OVA/IL-33–induced increases in lung DCs. (A) Timeline of mediator administration to the lung. Mice were treated intranasally with OVA/IL-33 on days 1 and 2. Control mice were treated with OVA (without IL-33) on the same schedule. In the IP-OVA/IL-33 group, mice were treated with STAT6-IP (or control saline), 1 d before and 1 h before each of two OVA/IL-33 administrations. In the OVA/IL-33-IP group, STAT6-IP was delivered on days 3 and 4 after OVA/IL-33 delivery on days 1 and 2. All mice were sacrificed on day 5. Numbers of CD11c^+^MHCII^+^ DCs in (B) the MLNs and (C) the lungs. (D) Numbers of CD11b^+^ cDCs (left), moDCs (middle), and CD103^+^ cDCs in the lungs. Symbols represent individual mice and data are presented as mean ± SEM. Data are from one experiment representative of two with 4 mice/group. Outcomes were assessed by 1-way analysis of variance, followed by Sidak’s multiple comparison test. *P < 0.05, **P < 0.01, ****P < 0.0001.

We investigated the effect of STAT6-IP delivery to the lung at two different time points—before and directly after priming through OVA/IL-33 delivery to the lung—on DC activation and migration to the MLNs; we also examined the lung inflammatory responses in these mice. Briefly, mice were treated acutely with OVA alone as a negative control^10^^,^^20^^,^^21^ or in the presence of IL-33, without or with STAT6-IP, which was delivered before (IP-OVA/IL-33) or directly after (OVA/IL-33-IP) sensitization as shown in Fig. 1A. A total of 72 h after the second OVA/IL-33 treatment (on day 5), lungs and MLNs were collected to examine effects of STAT6-IP on lung inflammation and DC activation and migration to the MLNs. The gating strategy used to characterize different DC populations is shown in Fig. S1A and B. OVA/IL-33 induced a significant increase in the absolute number of DCs identified as CD11c^+^MHCII^+^ in the MLNs (∼4.4 × 10^4^) (Fig. 1B) and lungs (∼1.8 × 10^6^) (Fig. 1C), which were significantly reduced to ∼1.0 × 10^4^ in the MLNs and ∼0.6 × 10^6^ in the lungs, in mice treated with STAT6-IP, either before or after OVA/IL-33 priming (Fig. 1B, C). STAT6-IP delivery inhibited CD11b^+^ cDCs and moDCs as well as CD103^+^ cDCs, whether delivered before or after OVA/IL-33 priming, in both the lung (Fig. 1D) and the MLNs (Fig. S1C). Although CD11b^+^ moDCs represented one of the major DC subsets in the lung, only minor accumulation of those cells was observed in the MLNs, consistent with data from Plantinga et al.,^22^ who showed that, in contrast to CD11b^+^ cDCs, moDCs exhibit poor migratory capacity in response to house dust mite. In addition, in OVA/IL-33–treated mice, compared with CD11b^+^ cDCs, far fewer CD103^+^ cDCs were recruited into the lung and MLNs (Fig. 1D;  Fig. S1C), also in agreement with previous studies.^7^^,^^22^

**Figure 2:**
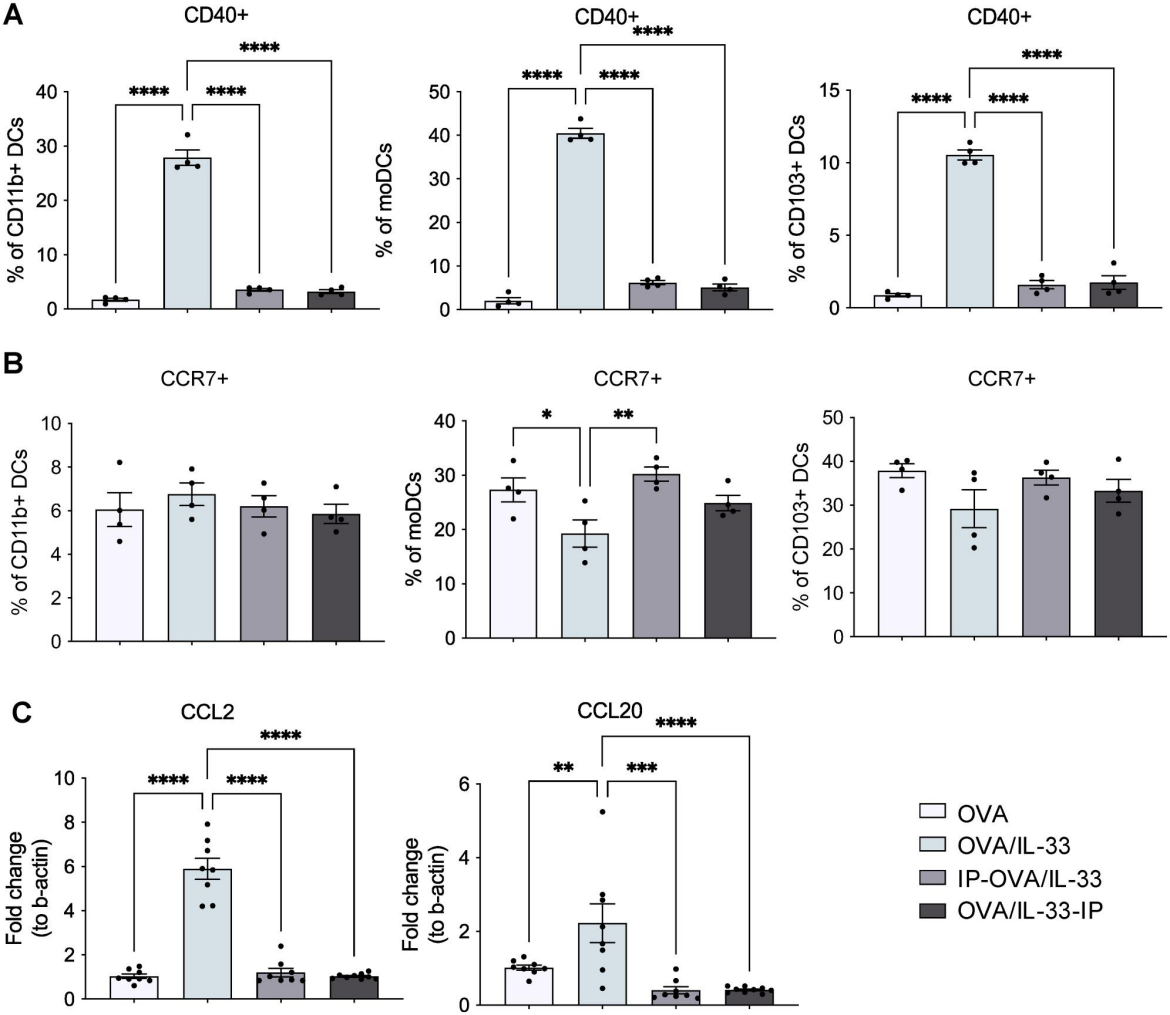
STAT6-IP inhibits OVA/IL-33–induced DC activation in the lung. Mice were treated as described in Fig. 1A and the frequency of lung DCs expressing (A) CD40 or (B) CCR7 within each DC subset (percent parent) were quantified. CD11b^+^ cDCs (left), moDCs (middle), and CD103^+^ cDCs were examined. (C) Relative mRNA levels of CCL2 (left) and CCL20 (right) in the lung. Symbols represent individual mice and data are presented as mean ± SEM. Data in panel A are from 1 experiment representative of 2 with 4 mice per group. Data in panel C are from the combination of 2 independent experiments (n = 4 or 5 mice per group in each experiment). Outcomes are presented as mean ± SEM assessed by 1-way analysis of variance followed by Sidak’s multiple comparison test. *P < 0.05, **P < 0.01, ***P < 0.001, ****P < 0.0001.

The CD40 costimulatory molecule promotes CD4^+^ T cell activation and differentiation through binding to its ligand CD40L (CD154) expressed by T cells.^23^ Therefore, the frequency of CD40-expressing DCs within each subtype was quantified as was their inhibition by STAT6-IP. In the lung, in response to OVA/IL-33, the frequency of CD11b^+^ cDCs and moDCs that upregulated CD40 was dramatically increased, whereas that of CD103^+^ cDCs was of much lower magnitude (Fig. 2A); moreover, each of these subsets was significantly reduced by STAT6-IP, whether delivered before or after OVA/IL-33 (Fig. 2A). In the MLNs, the frequency of CD11b^+^ cDCs and moDCs expressing CD40 was significantly reduced by STAT6-IP, though the magnitude of inhibition was much greater on moDCs (Fig. S1D), and STAT6-IP had no effect on CD40-expressing CD103^+^ cDCs in the MLNs (Fig. S1D).

The propensity for DC migration was also examined in these mice through quantification of CCR7, which is required for localization of DCs to the MLNs.^9^ Interestingly, only subtle changes in the proportion of CCR7-expressing DCs were found in the lung (Fig. 2B), and no differences were detected in the MLNs of mice treated with OVA/IL-33 (Fig. S1E), alone or with STAT6-IP. CCR7 MFI also did not differ in DCs in the lung or MLNs (data not shown).

CCL2 and CCL20 are the two main chemokines that promote recruitment of monocytes and immature DC to the site of inflammation.^22^^,^^24–27^ Levels of messenger RNA (mRNA) encoding CCL2 and CCL20 were significantly increased in the lungs of mice treated with OVA/IL-33, and this increase was reduced significantly by STAT6-IP (Fig. 2C). These data suggest that intranasal delivery of STAT6-IP before or directly after OVA/IL-33 delivery reduced DC recruitment and activation in the lungs to effectively reduce DC migration to the MLNs. However, STAT6-IP did not appear to target a particular DC subset, providing evidence that inhibition of DCs may be an indirect effect of STAT6-IP.

### STAT6-IP inhibitory activity is associated with diminished ILC2 expansion and cytokine production

**Figure 3:**
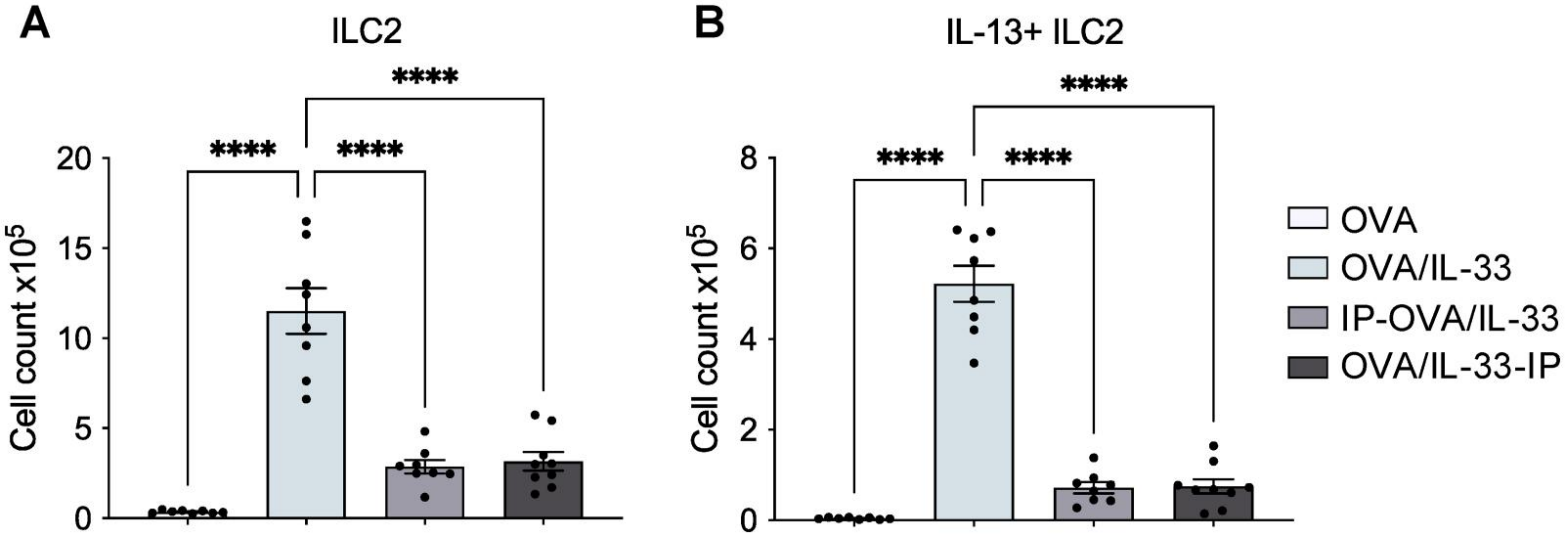
STAT6-IP inhibitory activity is associated with diminished ILC2 expansion and cytokine production. Mice were treated as described in Fig. 1A. ILC2s were gated as Lin^−^, lymphoid cells that were CD127^+^Thy1.2^+^KLRG1^+^ST2^+^. (A) Total numbers of lung ILC2s. (B) Total numbers of ILC2s expressing IL-13. Symbols represent individual mice. Data are the combination of 2 independent experiments using 4–5 mice per group in each experiment. Outcomes are presented as mean ± SEM assessed by 1-way analysis of variance followed by Sidak’s multiple comparison test. ****P < 0.0001.

ILC2-derived IL-13 can promote DC activation and migration to the MLNs,^8^ and we previously demonstrated that STAT6-IP potently inhibits IL-33–induced ILC2 expansion and cytokine production in the lung.^13^ Thus, we examined ILC2s as a possible target for STAT6-IP. We focused on KLRG1^+^ ILC2s, because our experiments used male mice, in which the majority of ILC2s express KLRG1.^15^^,^^28^^,^^29^ While delivery of OVA/IL-33 induced a significant increase in the number of KLRG^+^ ILC2s as well as ILC2s expressing IL-13, these were both reduced in mice treated with STAT6-IP, even when delivered after OVA/IL-33 (Fig. 3A, B). To better understand the mechanism by which STAT6-IP retained activity when delivered after OVA/IL-33 priming, we examined ILC2 responses in mice 24 h after administration of OVA/IL-33, the time of the first STAT6-IP delivery in the OVA/IL-33-IP group, and we compared those with ILC2 responses in mice at the 72 h time point, both with and without STAT6-IP. The timing of STAT6-IP delivery and assessment of lung ILC2 responses is shown in Fig. S3A. Mice were treated with OVA alone or with OVA/IL-33 on days 1 and 2, after which mice were sacrificed on either day 3 (24 h later) or, alternatively, treated with STAT6-IP or saline on days 3 and 4 and sacrificed on day 5 (72 h later). Our data show that after 24 h, proliferation of ILC2s had not yet occurred, though there was a modest (yet insignificant) increase in the number of cytokine-producing ILC2s in OVA/IL-33–treated mice (Fig. S3B–D). By 72 h, on the other hand, expansion of cytokine producing ILC2 was both significantly increased, and sensitive to STAT6-IP inhibition (Fig. S3B–D). Together, these data suggest that OVA/IL-33–induced ILC2 expansion did not occur immediately after OVA/-IL-33 exposure, in line with our published findings^15^ and those of others,^30^^,^^31^ and link STAT6-IP inhibition of ILC2 expansion and IL-13 production to reduced DC responses in the lung and MLNs.

### STAT6-IP inhibits OVA/IL-33–induced CD4^+^ Th2 differentiation in the MLNs

**Figure 4:**
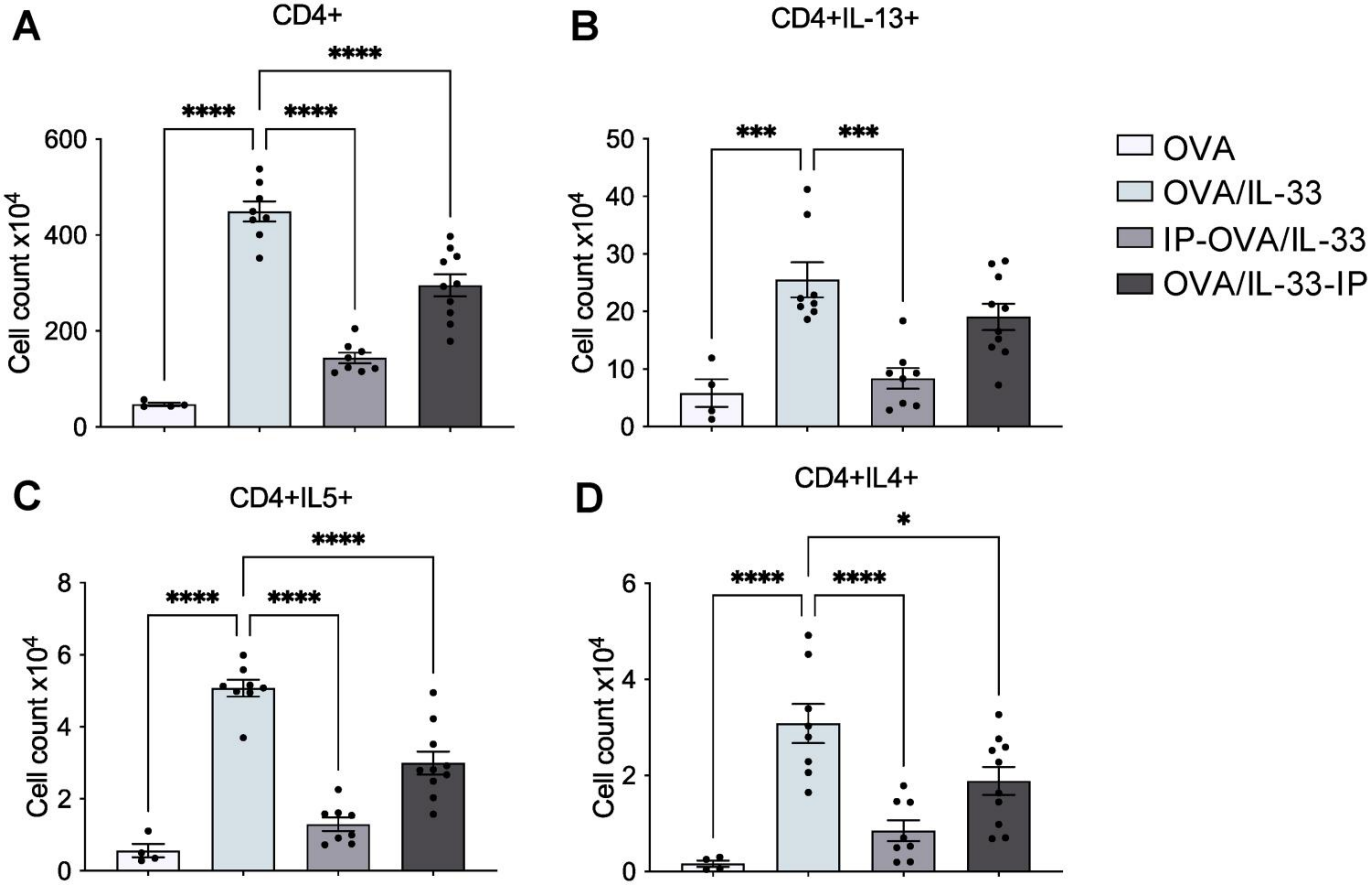
STAT6-IP inhibits OVA/IL-33–induced CD4^+^ Th2 differentiation in the MLNs. Mice were treated as indicated in Fig. 1A, except they were euthanized on day 8 as described in the methods and CD4^+^ T cells and their associated cytokine production in the MLNs quantified by flow cytometry. (A) Total numbers of CD4^+^ T cells as well as those producing (B) IL-13, (C) IL-5, and (D) IL-4 were quantified. Symbols represent individual mice. Data are pooled from 2 independent experiments using 2 to 5 mice per group in each experiment. Outcomes are presented as mean ± SEM assessed by 1-way analysis of variance, followed by Sidak’s multiple comparison test. *P < 0.05, ***P < 0.001, ****P < 0.0001.

Because DCs, in particular CD11b^+^ cDCs, are critical in Th2 priming,^7^^,^^22^ we hypothesized that modulation of DCs by STAT6-IP would reduce Th2 differentiation induced by OVA/IL-33. Mice were treated as in Fig. 1A (see also methods) and euthanized on day 8 in order to quantify total CD4^+^ T cells (Fig. 4A), as well as those expressing IL-13, IL-4, or IL-5 in the MLNs (Fig. 4B–D). Our data show that OVA/IL-33 induced robust expansion of CD4^+^ T cells as well as cytokine-producing Th2 cells in the MLNs, both of which were effectively reduced by STAT6-IP (Fig. 4A–D). Interesting, while STAT6-IP reduced DC in the MLNs to the same extent whether delivered before or after acute OVA/IL-33 treatment (Fig. S1B), expansion and cytokine production from CD4^+^ T cells were more efficiently inhibited by delivery of STAT6-IP before OVA/IL-33 priming, suggesting that the quality of antigen presentation and/or T cell activation by DCs in these groups differed.

### STAT6-IP delivery at OVA/IL-33–induced priming inhibits allergic airways disease induced upon secondary OVA challenge

**Figure 5:**
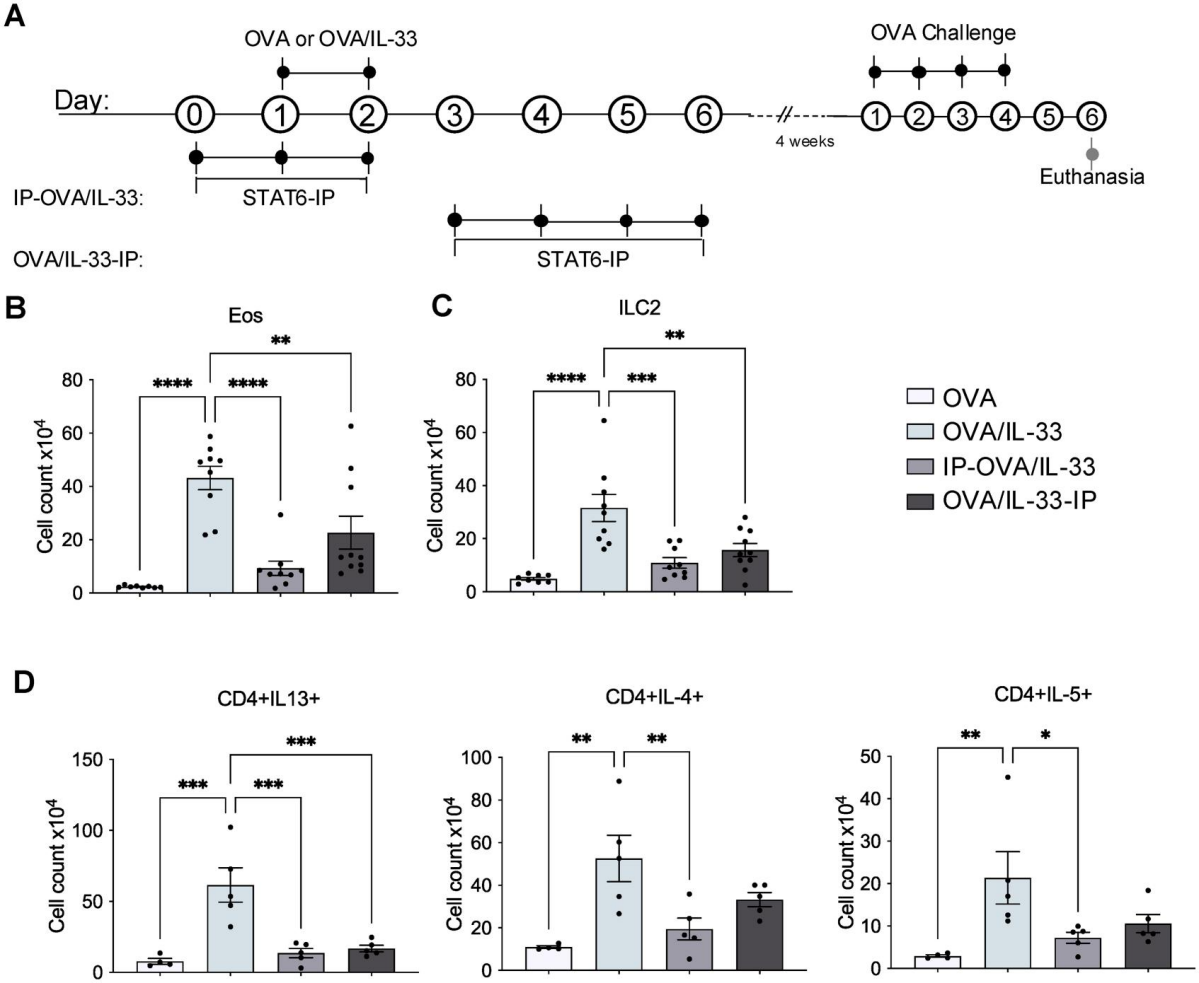
STAT6-IP delivery at the time of OVA/IL-33 priming inhibits type 2 lung inflammation induced upon secondary OVA challenge. (A) Mice were treated with OVA/IL-33 alone or with STAT6-IP either before or after sensitization as described in Fig. 1A, except STAT6-IP was given for 4 d in the OVA/IL-33-IP group. After 4 wk, mice were challenged with OVA once per day for each of 4 d and were euthanized 48 h later. Lung cells were analyzed by flow cytometry. Absolute count of lung (B) eosinophils, (C) ILC2s, and (D) CD4^+^ T cells producing IL-13, IL-4, or IL-5. Symbols represent individual mice and data are presented as mean ± SEM. Data in panels B and C are from the combination of 2 independent experiments using 4 or 5 mice per group in each experiment. Data in panel D are from one experiment representative of 2 with 4 or 5 mice/group. Outcomes were assessed by 1-way analysis of variance, followed by Sidak’s multiple comparison test. *P < 0.05, **P < 0.01, ***P < 0.001, ****P < 0.0001.

**Figure 6:**
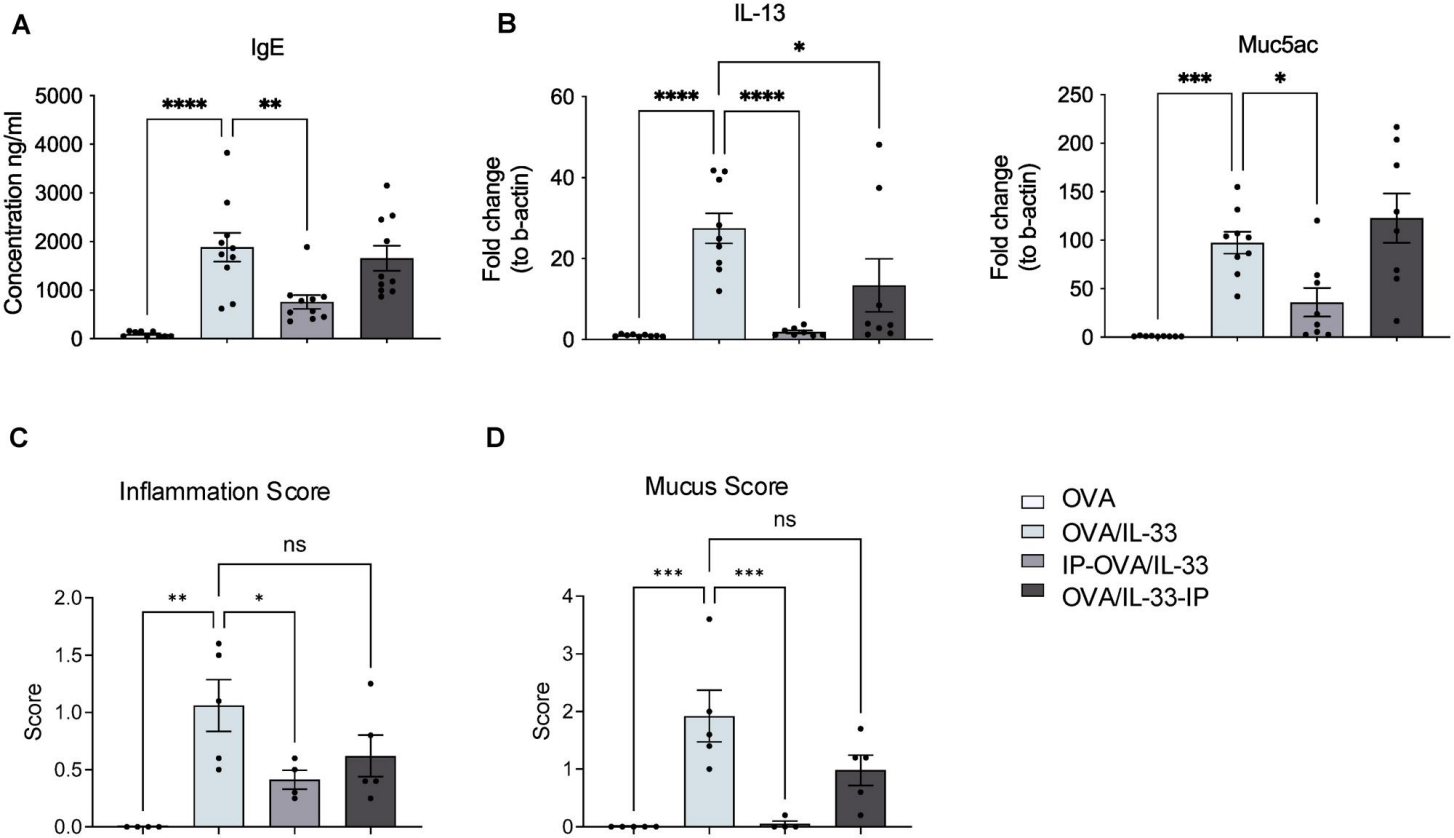
STAT6-IP delivery at the time of OVA/IL-33 priming reduces OVA-specific IgE and *MUC5AC* and *IL-13* mRNA levels induced upon secondary OVA challenge. Mice were treated as described in Fig. 5A. (A) Serum levels of OVA-specific IgE were quantified. Levels of (B) *IL-13* and *MUC5AC* mRNA were calculated with the ΔΔCT method, normalized to *β-actin*, and presented relative to levels in OVA-treated control mice. (C, D) Semi-quantitative analysis of (C) tissue inflammation and (D) mucus-producing cells. Histological changes were quantified by an individual, blinded to the groups, using a scale from 0 to 2 for tissue inflammation and 0 to 5 for mucus. Symbols represent individual mice and data are presented as mean ± SEM from the combination of two independent experiments using 4 or 5 mice per group in each experiment. Outcomes were assessed by 1-way analysis of variance, followed by Sidak’s multiple comparison test. *P < 0.05, **P < 0.01, ***P < 0.001, ****P < 0.0001.

To confirm that modulation of DC activation and Th2 differentiation by STAT6-IP at the time of antigen priming was associated with reduced Th2 adaptive immunity following OVA challenge, mice were treated with OVA/IL-33 alone or with STAT6-IP. After 4 wk, mice were challenged with OVA once per day for each of 4 d and euthanized 48 h after the last OVA challenge (Fig. 5A). Both lung eosinophils (Fig. 5B;  Fig. S4) and ILC2s (Fig. 5C) were significantly increased in OVA-challenged mice primed with OVA/IL-33, and each of these responses was significantly reduced in mice treated with STAT6-IP at the time of priming. Similar to our findings of robust Th2 differentiation in the MLNs at priming, OVA challenge led to a large increase in total CD4^+^ T cells (data not shown) as well as those expressing type 2 cytokines (Fig. 5D), all of which were inhibited by STAT6-IP treatment at the time of priming. Interestingly, levels of OVA-specific IgE, which were increased in OVA/IL-33–treated mice following OVA challenge, were significantly reduced by STAT6-IP delivery, but only when delivered before OVA/IL-33 priming (Fig. 6A). Similarly, the lung mRNA levels of *IL-13* and *MUC5AC* were increased in OVA/IL-33–treated mice following OVA challenge and most effectively reduced by STAT6-IP delivery before OVA/IL-33 priming (Fig. 6B, C). When STAT6-IP was delivered after OVA/IL-33 priming, levels of OVA-specific IgE and *MUC5AC* mRNA were not diminished. Altogether, these data demonstrate that STAT6-IP treatment at the time of OVA/IL-33 priming, effectively reduced most aspects of Th2 adaptive immunity induced upon secondary OVA challenge weeks later.

**Figure 7:**
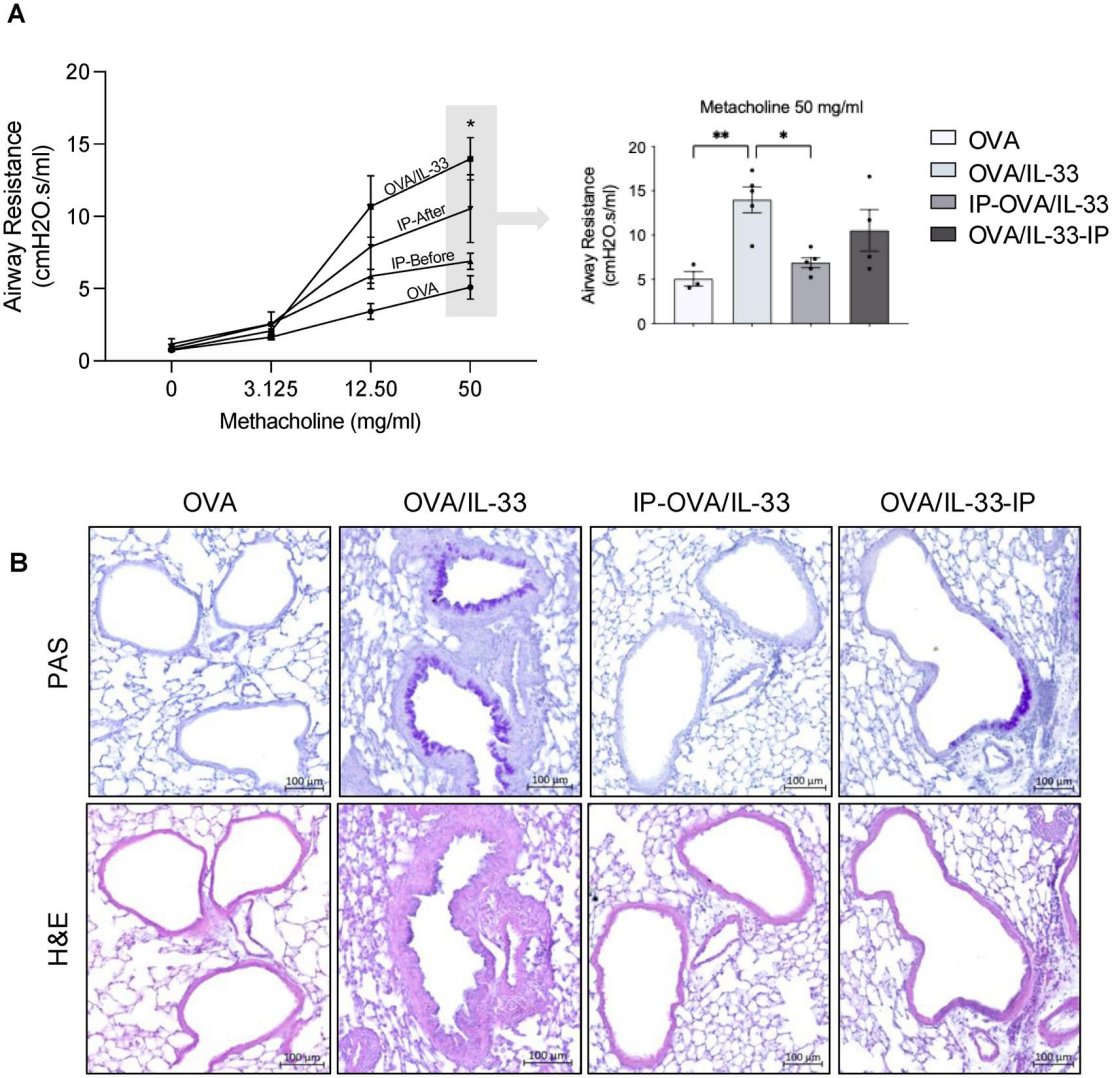
STAT6-IP delivery at OVA/IL-33–induced priming inhibits AHR, tissue inflammation, and mucus production induced upon secondary OVA challenge. Mice were treated as described in Fig. 5A. (A) Lung function was assessed with the flexiVent small animal ventilator and presented as total lung resistance in response to increasing doses of methacholine. (B) Periodic acid–Schiff (PAS) staining for mucus (top) and (C) hematoxylin and eosin (H&E) staining for peribronchial and perivascular inflammation were performed. Data in panel A are presented as mean ± SEM from 1 experiment using 4 or 5 mice per group. Outcomes were assessed by 2-way analysis of variance, followed by Tukey’s multiple comparison test. *P < 0.05, **P < 0.01. (B) Images shown are from 1 experiment and are representative of 4 or 5 mice per group in each of two independent experiments. Scale bar = 100 µm.

To confirm inhibitory activity of STAT6-IP on enhanced responses in OVA/IL-33 treated mice, we examined AHR, inflammation, and mucus levels by flexiVent and histology, respectively. Following OVA challenge, total lung resistance was significantly greater in OVA/IL-33–primed mice compared with mice sensitized and challenged with OVA (Fig. 7A). OVA/IL-33–primed mice challenged with OVA also exhibited abundant mucus production and inflammatory cell infiltration (Fig. 7B, C) around the airways. Each of these responses was reduced in mice treated with STAT6-IP before OVA/IL-33, but less so in mice that received STAT6-IP after OVA/IL-33 priming (Fig. 7A–C). Altogether, these data show that intranasal delivery of STAT6-IP, selectively at the time of Th2 priming, reduced allergic airways disease induced upon secondary OVA challenge several weeks later.

## Discussion

Data from our earlier work with STAT6-IP showed that intranasal administration of STAT6-IP reduced type 2 inflammation, though the targets of STAT6-IP were not identified.^32^^,^^33^ Our more recent work demonstrated that delivery of STAT6-IP prior to OVA/MDP priming impacted DCs and reduced type 2 airway inflammatory responses induced upon OVA challenge many weeks later.^10^ In addition to DCs, we hypothesized that STAT6-IP may have immunomodulatory effects on other innate immune cells present in the naïve lung. We have extended these findings and now show that STAT6-IP delivered at the time of OVA/IL-33 priming inhibits ILC2s as well as both cDCs and moDCs, at least in part through inhibition of DC chemokine production. STAT6-IP directly delivery after OVA/IL-33 priming coincided with inhibition of ILC2 expansion, and thus we speculate that the mechanism of action of STAT6-IP is, at least in part, reduced expansion of the ILC2 population, thereby effectively reducing ILC2-dependent events, including DC activation, as well as eosinophil influx and activation in the lung. In addition, the fact that STAT6-IP reduced lung DCs and their transit to the MLNs (in this study and in Lee et al.)^10^ provides evidence that the long-lasting inhibitory effects of STAT6-IP are due, at least in part, to the early modulation of DC responses that drive Th2 adaptive immunity. In this study, we focused on more precisely defining how STAT6-IP affected DCs during the innate immune response and how that provided protection during Th2 adaptive immunity induced upon secondary antigen challenge several weeks later.

DCs in the lung are a heterogeneous cell population, classified into moDCs and cDCs, which are further divided into CD103-expressing cDC1s and CD11b-expressing cDC2s. In mice, CD103^+^ cDCs are highly specialized in cross-presenting antigen to CD8^+^ T cells, whereas CD11b^+^ cDCs are more migratory and more efficient at presenting antigen on MHCII molecules and are therefore considered to be more efficient at inducing differentiation of CD4^+^ T cells.^34^^,^^35^ In this study, we also sought to determine if STAT6-IP exhibited inhibitory activity toward specific DC subsets. Our data show that the increase in CD11b^+^ cDCs in the lung and MLNs of OVA/IL-33–treated mice was significant and of greater magnitude compared with CD103^+^ cDCs and moDCs. Nevertheless, STAT6-IP exhibited potent inhibitory activity toward each of these DC subsets. CD40 signaling is important for DC maturation via upregulating MHCII, as well as the CD80 and CD86 costimulatory molecules, and is critical for the induction of Th2 cell differentiation.^23^^,^^36^^,^^37^ Our data show that in the lungs of OVA/IL-33 treated mice, the proportion of total CD11b^+^ cDCs and moDCs expressing CD40 was dramatically increased (∼30 fold), whereas in CD103^+^ cDCs, this increase was only ∼10 fold. Nevertheless, STAT6-IP, whether delivered before or after OVA/IL-33, reduced this to levels found in OVA-treated control mice, providing evidence that STAT6-IP inhibits activation of many DC subtypes. Moreover, these findings are compatible with published data showing that CD11b^+^ cDCs are the predominant inducers of Th2 responses in the lung.^7^^,^^22^

DC migration was also indirectly examined through quantification of CCR7 expression, which is required for localization of DCs to the MLNs. No significant differences were observed between mice treated with OVA/IL-33 and those treated with STAT6-IP. In fact, no differences in CCR7-expressing DCs in the MLNs were noted, even between OVA- and OVA/IL-33–treated mice. This raises the possibility that we have simply not detected the population of CCR7^+^ DCs that migrated into the MLNs, at least in part, because all of DC measurements were made at one time point: 72 h after the second OVA/IL-33 administration. Moreover, while we originally hypothesized that STAT6-IP would reduce DC migration to the MLNs, and that the proportion of CCR7-expressing DCs in the MLNs would be reduced, the dramatic increase in DCs in the lungs of OVA/IL-33–treated mice and, in turn, their dramatic inhibition by STAT6-IP suggested that the changes in DC numbers in the MLNs may simply reflect smaller numbers of DCs in the lung.

In response to infection or allergen inhalation, lung epithelial cells secrete chemokines, including CCL2 and CCL20. CCL2 drives recruitment of monocytes into the lungs where they differentiate into moDCs and produce large amounts of chemokines to recruit Th2 cells into the lung.^22^^,^^24^^,^^26^ CCL20, via its receptor CCR6, attracts immature DCs to sites of inflammation.^25^^,^^27^ Thus, the reductions in CCL2 and CCL20 by STAT6-IP likely reduced recruitment of both moDCs and precursors of cDCs into the lung, subsequently reducing the number available to be recruited into the MLNs. In addition, Grunig and colleagues demonstrated that administration of recombinant IL-13 to the lung increases MHCII and CD40 on lung CD11c^+^ (dendritic) cells, in a manner that is dependent upon IL-4Rα, one subunit of the cognate IL-13 receptor.^38^ More recent data show that activated ILC2s proliferate and secrete large amounts of IL-13, which induces activation and migration of DCs to the MLNs where they induce Th2 differentiation.^8^^,^^39^ Thus, our data implicate IL-33–induced IL-13 production by ILC2s in the increases in DC activation and migration to the MLNs. It is certainly possible that the dramatic inhibition of IL-13–producing ILC2s by STAT6-IP also reduced DC activation and migration from the lungs to MLNs.

In agreement with the STAT6-IP–dependent inhibition of DC recruitment and activation, we also show that STAT6-IP reduced OVA/IL-33–induced Th2 differentiation: total and cytokine-producing CD4^+^ Th2 cells in the MLNs were significantly reduced by STAT6-IP, whether delivered before or after OVA/IL-33 priming. Nevertheless, greater inhibition was observed when STAT6-IP was delivered before OVA/IL-33 priming even though the numbers of DCs were equally inhibited by STAT6-IP when delivered at each time point. One possible explanation is that these DCs exhibit phenotypic differences and therefore differentially regulate Th2 differentiation. Examining other costimulatory molecules that promote Th2 differentiation (e.g. CD86, CD80, and OX40L) may provide further insight.

We hypothesized that the early effect of STAT6-IP on Th2 priming would inhibit Th2 adaptive immunity and allergic airways disease induced upon OVA challenge weeks later. Because delivery of STAT6-IP prior to OVA/IL-33 priming more effectively reduced Th2 differentiation in the MLNs, we predicted more effective inhibition with this delivery regimen, which was the case. Delivery of STAT6-IP prior to OVA/IL-33 effectively reduced type 2 inflammatory responses, including AHR, while inhibitory activity was more modest when STAT6-IP was delivered after OVA/IL-33 priming, even while ILC2s and IL-13–producing CD4^+^ T cells were equally reduced. Inhibition in the levels of OVA-specific IgE provided evidence that follicular helper T cells may also have been affected by STAT6-IP delivery before OVA/IL-33 sensitization,^40^ though further work is required to define the precise mechanism(s) by which STAT6-IP reduces OVA-specific IgE.

Oliphant et al.^5^ showed that MHCII-expressing ILC2s have the ability to present antigen to CD4^+^ T cells leading to IL-2 production (by T cells), which in turn promotes ILC2 proliferation and IL-13 production. Upon OVA challenge, we observed an increase in the number of IL13^+^ ILC2s and CD4^+^ T cells in OVA/IL-33–sensitized mice, suggesting there may be crosstalk between ILC2s and T cells in which ILC2s promote Th2 differentiation and cytokine production and T cells support ILC2 proliferation and activation. In fact, recent studies have revealed that ILC2s not only are involved in the initiation of type 2 immunity, but also can enhance the adaptive immune responses.^6^^,^^41^^,^^42^ Data from Martinez-Gonzalez et al.^43^ showed that ILC2s, previously activated by IL-33, have memory-like activity and, upon secondary reactivation, have enhanced ability to drive Th2 differentiation in the MLNs. Our own data show that OVA/IL-33–experienced ILC2s remain elevated 1 mo after activation.^14^ Our data here suggest STAT6-IP may effectively interrupt this memory-like response in ILC2s, consistent with our data showing the loss of this activity in STAT6 knockout mice.^14^

## Supplementary Material

vlag012_Supplementary_Data

## Data Availability

The datasets used and/or analyzed during the current study are available from E.D.F. on reasonable request.
