# Supplementary material for: STAT6-IP–dependent inhibition of type 2 innate and Th2 adaptive immunity in the murine lung
Source: Immunohorizons. 2026 Mar 29;10(3):vlag012. doi: 10.1093/immhor/vlag012 (PMC13033214; doi:10.1093/immhor/vlag012)

## Supplemental Table 1

### Dendritic cell panel

| Antibody | Conjugation | Clone | Company |
|---|---|---|---|
| CD19 | PE | eBio1D3 | eBioscience |
| CD3e | PE | 145-2C11 | Biolegend |
| CD49b | PE | DX5 | BD |
| Ly6G | PE | 1A8 | Biolegend |
| CD11b | BV605 | M1/70 | Biolegend |
| CD11c | APC | N418 | Biolegend |
| F4/80 | FITC | BM8 | eBioscience |
| CCR7 (CD197) | BV786 | 4B12 | BD |
| CD103 | PeCy7 | 2E7 | Biolegend |
| FceR1 | PerCP-eFluor710 | MAR-1 | eBioscience |
| CD45 | AlexaFluor700 | 30-F11 | Biolegend |
| MHCII | BV510 | M5/114.15.2 | Biolegend |
| B220 | BV650 | RA3-6B2 | BD |
| CD8a | BUV737 | 53-6.7 | BD Bioscience |
| CD40 | BUV395 | Mar-23 | BD |

## Supplemental Table 2

### A - Eosinophil panel

| Antibody | Conjugation | Clone | Company |
|---|---|---|---|
| Siglec-F | PE | E50-2440 | BD |
| Ly6G | AlexaFluor700 | 1A8 | Biolegend |
| CD45.2 | BUV395 | 104 | BD |
| CD11c | AF488 | N418 | Lifetech |
| CD11b | PeCy7 | M1/70 | eBioscience |
| F4/80 | APC | BM8 | eBioscience |

### B - T cell panel

| Antibody | Conjugation | Clone | Company |
|---|---|---|---|
| CD4 | FITC | RM4-5 | eBioscience |
| CD3 | V500 | 500A2 | BD |
| CD8 | PerCP-Cy5.5 | 53-6.7 | Lifetech |
| CD45.2 | BUV395 | 104 | BD |
| IL-13 | PE | eBio13A | eBioscience |
| IL-5 | BV421 | TRFK5 | eBioscience |
| IL-4 | APC | 11B11 | eBioscience |

## Supplemental Table 3

## ILC2 panel

| Antibody | Conjugation | Clone | Company |
|---|---|---|---|
| CD45R | PE | RA3-6B2 | Lifetech |
| CD3e | PE | 145-2C11 | Biolegend |
| CD49b | PE | DX5 | BD |
| Ly6G | PE | 1A8 | Biolegend |
| TCRyD | PE | GL3 | BD |
| CD11c | PE | N4/8 | Biolegend |
| CD11b | PE | M1/70 | eBioscience |
| FceR1a | PE | MAR1 | Biolegend |
| Thy1.2 (CD90.2) | EF450 | 53-2.1 | eBioscience |
| CD127 (IL7Ra) | PeCy7 | A7R34 | Biolegend |
| KLRG1 | BV605 | 2F1/KLRG1 | Biolegend |
| MHCII | BV510 | M5/114.15.2 | Biolegend |
| ST2 | PerCP-eFluor710 | RMST2-2 | Biolegend |
| CD45.2 | BUV395 | 104 | BD |
| IL-13 | AF488 | eBio13A | Lifetech |
| IL-5 | APC | TRFK5 | BD |

## Supplemental Figure Legends

**Figure S1. STAT6-IP inhibits recruitment, activation of lung DCs and their migration to the MLN. (A)** Flow cytometry gating strategy to identify different DC subsets in the lung and MLN. Cells were gated first on live cells and singlets, then on lin-F4/80- cells, followed by CD45+ cells. Total DCs were identified as CD11c+MHCII+. Within the DC population, cDC1s were identified as CD103+CD11b-. MAR1 was used to discriminate between moDCs (MAR1+) and CD11b+ cDC2s (MAR1-). The CD40 co-stimulatory and CCR7 migratory molecules were gated from each DC population. **(B)** FMO controls for MHCII, MAR1, CD40, CCR7. **(C)** Numbers of cDC2s, moDCs and cDC1s in the MLN. The frequency of DCs expressing **(D)** CD40 or **(E)** CCR7 within each DC subset. Symbols represent individual mice and data are presented as mean ± SEM. Data are from one experiment representative of two with 4 mice/group. Outcomes were assessed by one-way ANOVA, followed by Sidak's multiple comparison test. *p<0.05, **p<0.01, ***p<0.001, ****p<0.0001.

**Figure S2. Flow cytometry gating strategy to identify ILC2s and their cytokine production.** After removing debris, cells were gated on singlets and live cells, then gated on CD45+Lin-. ILC2s were identified as lymphoid like, Lin-CD127+Thy1.2+ST2+, expressing KLRG1 or not. ILC2s producing IL-13 and/or IL-5 were identified. FMO controls for IL-5 and IL-13 are shown.

**Figure S3. Administration of STAT6-IP post OVA/ IL-33 priming interrupts ILC2 expansion. (A)** Balb/c mice were treated intranasally with IL-33 and/or OVA on day 1 and day 2. One group of mice was sacrificed 24h later. Another group was treated with STAT6-IP or control saline on day 3 and day 4 and sacrificed on day 5. **(B)** Total ILC2s as well as those producing **(C)** only IL-13 or **(D)** only IL-5 were quantified. Symbols represent individual mice and data are presented as mean ± SEM from one experiment using 3-5 mice per group. Outcomes were assessed by one-way ANOVA, followed by Sidak's multiple comparison test. *p<0.05, **p<0.01, ***p<0.001.

**Figure S4. Flow cytometry gating strategy to identify eosinophils.** After removing debris, cells were gated on singlets and live cells. $Ly6G^{hi}$ cells were removed and total eosinophils identified as $CD45^{+}Ly6G^{-/int}SiglecF^{+}CD11c^{-/lo}$

## Supplemental Figure 1

(A)

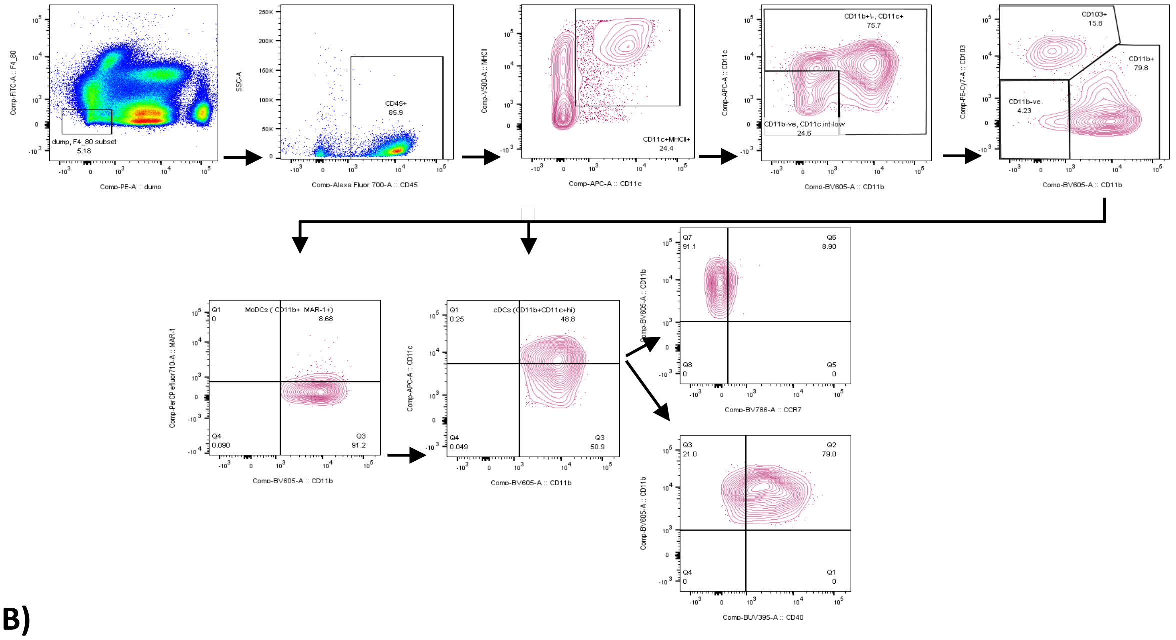


(B)

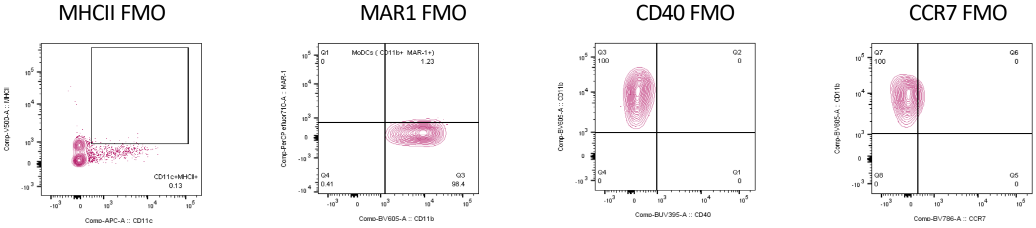


(C)

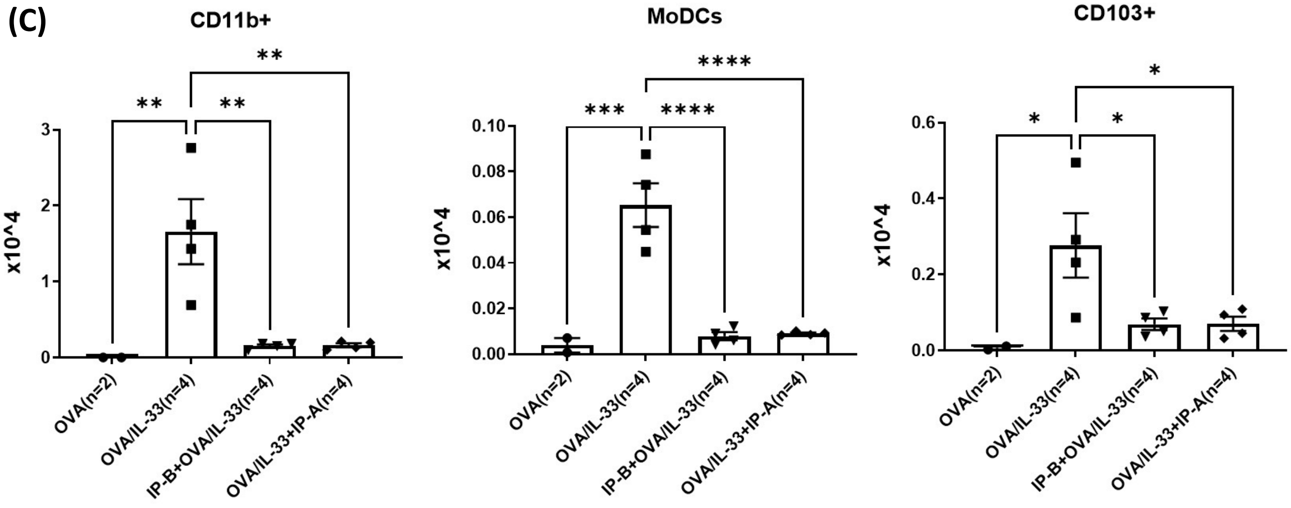

(D)

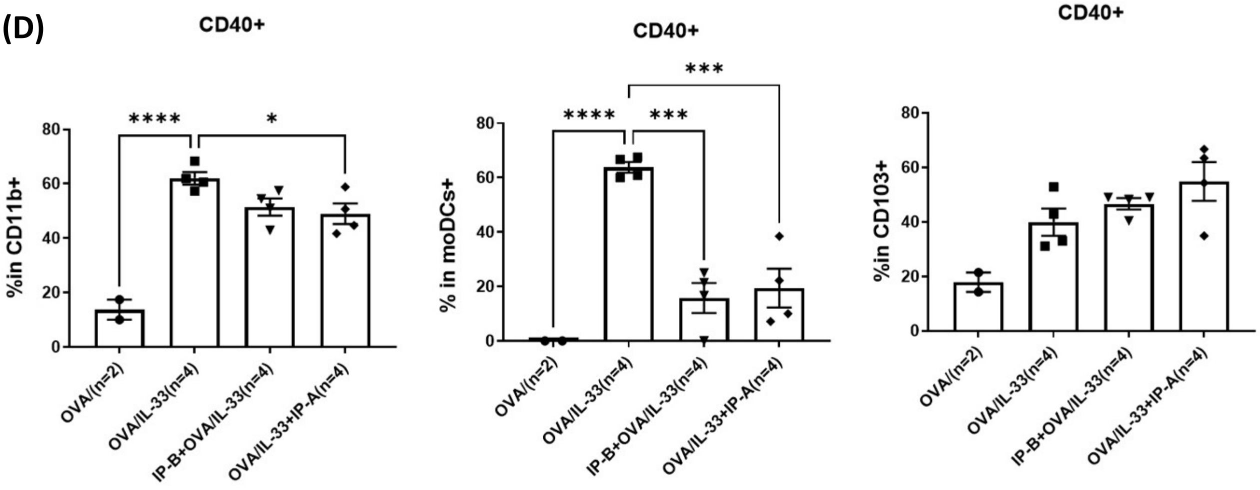
CD40+
%in CD11b+
****
*
CD40+
% in moDCs+
****
***
***
CD40+
%in CD103+
OVA/(n=2)
OVA/IL-33(n=4)
IP-B+OVA/IL-33(n=4)
OVA/IL-33+IP-A(n=4)

(E)

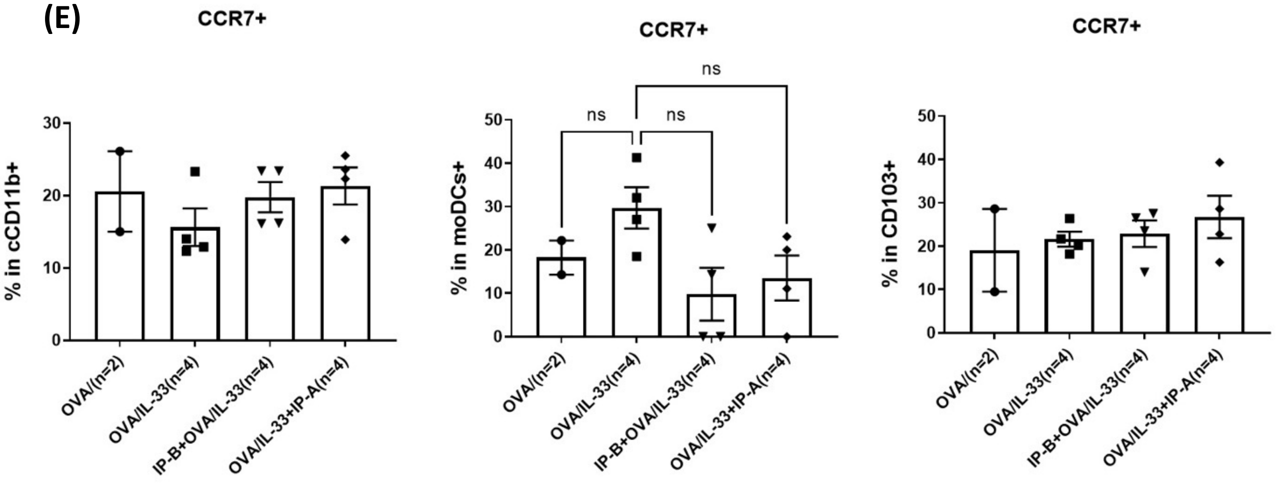
CCR7+
% in cCD11b+
CCR7+
% in moDCs+
ns
ns
ns
CCR7+
% in CD103+
OVA/(n=2)
OVA/IL-33(n=4)
IP-B+OVA/IL-33(n=4)
OVA/IL-33+IP-A(n=4)

## Supplemental Figure 2

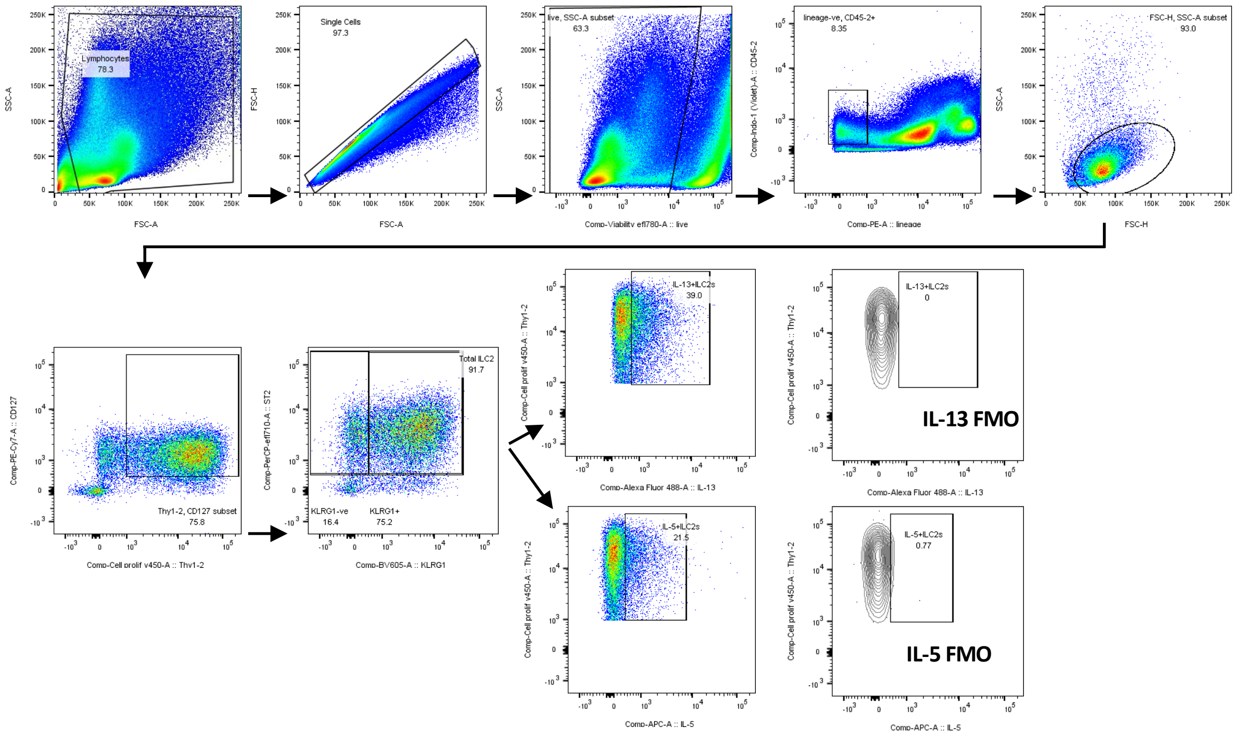

## Supplemental Figure 3

**(A)**

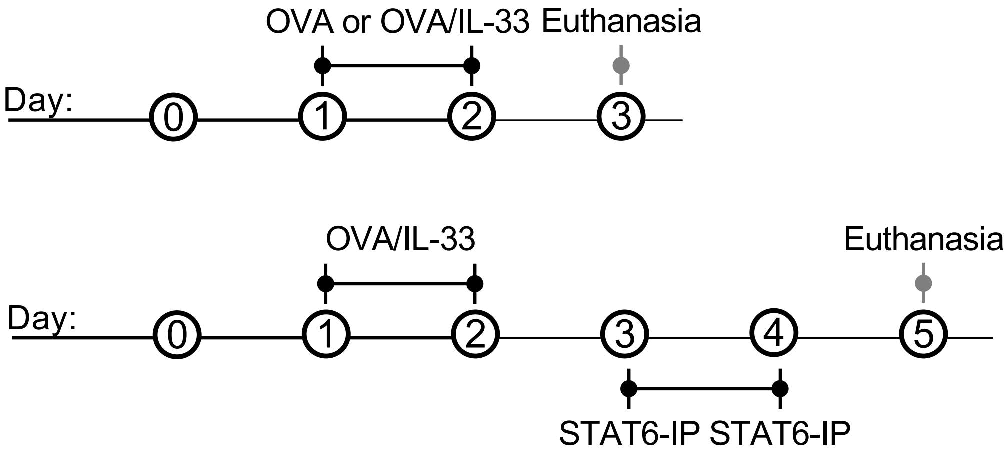


**(B)**

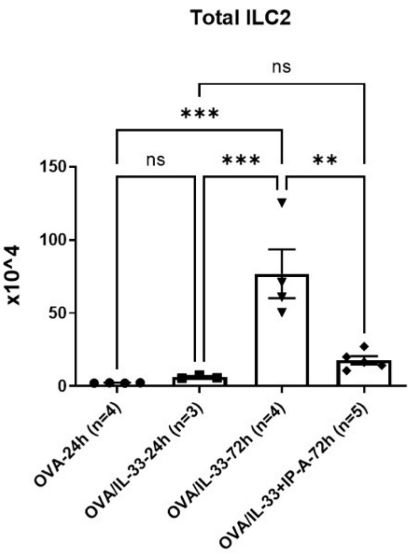


**(C)**

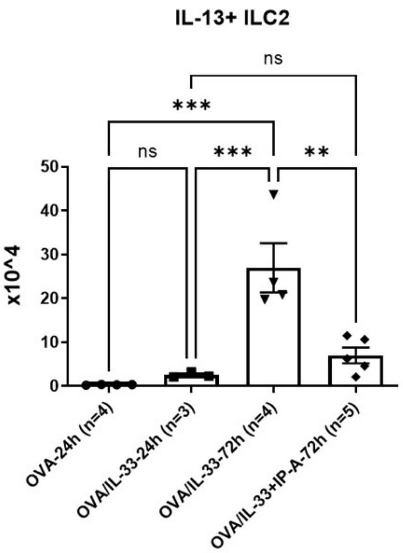


**(D)**

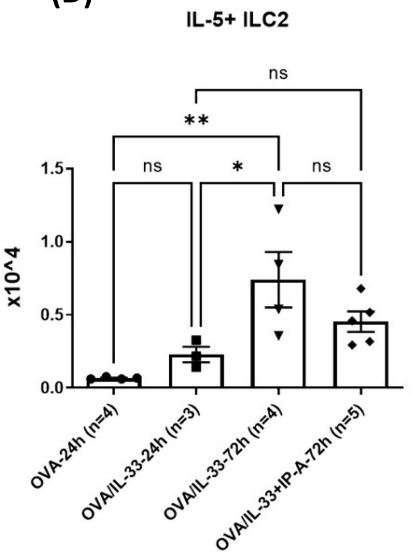

# Supplemental Figure 4

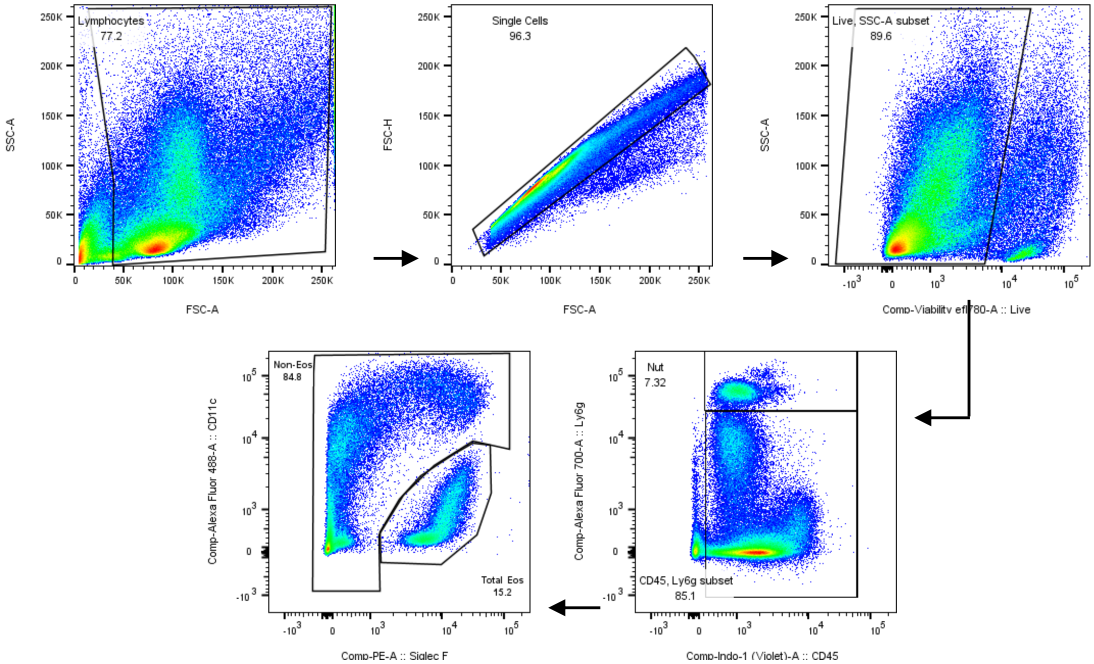

Supplement: vlag012_Supplementary_Data [file vlag012_supplementary_data.pdf]
